# Systemischer Pruritus: Was gibt es Neues in Diagnostik und Therapie?

**DOI:** 10.1007/s00105-022-05027-z

**Published:** 2022-06-30

**Authors:** M. Brand, A. E. Kremer

**Affiliations:** 1 Medizinische Klinik D, Universitätsklinikum Münster, Münster, Deutschland; 2 Klinik für Gastroenterologie und Hepatologie, UniversitätsSpital Zürich, Rämistr. 100, 8091 Zürich, Schweiz

**Keywords:** Cholestase, Chronische Niereninsuffizienz, Juckreiz, Leber, Myeloproliferative Neoplasien, Cholestasis, Chronic kidney disease, Itching/pruritus, Liver, Myeloproliferative neoplasms

## Abstract

**Hintergrund:**

Chronischer Pruritus ist ein häufiges Symptom zahlreicher internistischer Erkrankungen. Insbesondere sind Patienten mit chronischer Niereninsuffizienz, hepatobiliären Erkrankungen und myeloproliferativen Neoplasien betroffen.

**Ziel der Arbeit:**

Dieser Übersichtsartikel soll einen Überblick über die laborchemische und bildgebende Diagnostik sowie aktuelle und neuartige Therapieansätze des Pruritus systemischer Erkrankungen geben.

**Material und Methoden:**

Es erfolgte eine ausführliche PubMed-Recherche.

**Ergebnisse:**

Zur Abklärung des chronischen Pruritus wird eine Stufendiagnostik empfohlen, die sich an der Häufigkeit der mit Pruritus assoziierten Erkrankungen orientiert. Eine Basisdiagnostik ermöglicht dabei eine kostengünstige und gezielte Abklärung auf hausärztlicher Ebene. Aktuelle topische und medikamentöse Therapieempfehlungen des Pruritus bei chronischer Niereninsuffizienz, hepatobiliären Erkrankungen und myeloproliferativen Neoplasien sowie selteneren Ursachen wurden übersichtlich zusammengefasst. Daneben werden neuartige Therapieansätze wie der κ‑Opioidrezeptor-Agonist Difelikefalin, Bezafibrat, Inhibitoren des „ileal bile acid transporter“ (IBAT) und des JAK(Januskinase)-STAT(„signal transducers and activators of transcription“)-Signalweges aufgezeigt.

**Diskussion:**

Chronischer Pruritus bei systemischen Erkrankungen kann eine diagnostische Herausforderung darstellen. Eine Stufendiagnostik erleichtert die Identifikation der zugrunde liegenden Erkrankung. Ein verbessertes pathophysiologisches Verständnis hat zu ersten zugelassenen Therapieoptionen bei „chronic kidney disease“-assoziiertem und hepatischem Pruritus geführt.

## Abstract (en)

**Background:**

Chronic pruritus is a common symptom of various systemic diseases. In particular, patients with chronic renal failure, hepatobiliary diseases, and myeloproliferative neoplasms are affected.

**Objectives:**

The purpose of this review is to provide an overview of laboratory chemistry and imaging diagnostics as well as current and novel therapeutic approaches to pruritus of systemic diseases.

**Materials and methods:**

An extensive PubMed search was performed.

**Results:**

To clarify the cause of chronic pruritus, a step-by-step diagnosis is recommended, which is based on the frequency of pruritus-associated diseases. A basic diagnosis enables a cost-effective and targeted clarification at the level of a general practitioner. Current topical and drug therapy recommendations of pruritus in chronic renal failure, hepatobiliary diseases, myeloproliferative neoplasms, and rarer causes are summarized. In addition, novel therapeutic approaches such as the κ‑opioid receptor agonist difelikefalin, bezafibrate, inhibitors of the ileal bile acid transporter (IBAT), and the JAK-STAT pathway are highlighted.

**Conclusions:**

Chronic pruritus in systemic diseases can be a diagnostic challenge. A staged diagnostic approach facilitates identification of the underlying disease. Improved pathophysiological understanding has led to the first approved therapeutic options for chronic kidney disease-associated and hepatic pruritus.

Neben klassischen Hauterkrankungen wie atopischer Dermatitis oder Psoriasis, leiden auch zahlreiche Patienten mit internistischen Krankheitsbildern an Pruritus. Aufgrund fehlender primärer Hautveränderungen kann die ätiologische Zuordnung herausfordernd sein. Der folgende Beitrag gibt einen Überblick über die gezielte laborchemische und bildgebende Diagnostik sowie neue Therapieansätze wie κ‑Opioidrezeptor-Agonisten, ileale Gallensalzwiederaufnahme(IBAT)-Hemmer und JAK(Januskinase)-STAT(„signal transducers and activators of transcription“)-Inhibitoren.

Chronischer Pruritus kann mild und tolerabel sein, aber auch moderate bis schwere Formen annehmen. Moderate bis schwerwiegende Intensitäten belasten die betroffenen Patienten häufig sehr und können deren Lebensqualität deutlich reduzieren. Vor allem quälender nächtlicher Pruritus kann gravierenden Schlafmangel verursachen, der Müdigkeit, Abgeschlagenheit, Depression und sogar Selbstmordgedanken zur Folge haben kann. Der Schweregrad des Pruritus kann im klinischen Alltag einfach mittels verbaler oder numerischer Ratingskala erhoben werden, wobei 0 „kein Pruritus“ und 10 dem „schwersten vorstellbaren Juckempfinden“ entspricht [19].

## Diagnostik

Besteht Pruritus auf primär läsionaler Haut, sind meist dermatologische Erkrankungen ursächlich wie entzündliche Dermatosen, infektiöse Dermatosen, Autoimmundermatosen, Genodermatosen sowie Neoplasien. Diagnostisch kommen bakteriologische, mykologische, allergologische und autoimmunserologische Untersuchungsverfahren zum Einsatz, die in der aktuellen S2k-Leitlinie übersichtlich dargestellt sind (Tab. 1). In unklaren Fällen wird häufig eine Hautbiopsie mit entsprechender Aufarbeitung benötigt.

**Table 1:** 

**Basisuntersuchungen**
*Labordiagnostik*
Blutsenkungsgeschwindigkeit (BSG) und C‑reaktives Protein (CRP)
Blutbild mit Differenzialblutbild, Ferritin
Bilirubin, Transaminasen (GPT [ALAT], GOT [ASAT]), Gammaglutamyl-Transferase (GGT), alkalische Phosphatase
Kreatinin, Harnstoff, errechnete glomeruläre Filtrationsrate (eGFR), K^+^, Urin (Streifentest)
Blutzucker nüchtern
Laktatdehydrogenase (LDH)
Thyreoidea-stimulierendes Hormon (TSH)
*Bei primären oder sekundären Hautveränderungen ggf.*
Bakteriologische/mykologische Abstriche
Hautbiopsie (Histologie, direkte Immunfluoreszenz, Elektronenmikroskopie)
Skabiesmilbennachweis
*Pruritus in der Schwangerschaft*
Bei auffälligem Hautbefund: dermatologische Untersuchung zum Ausschluss atopische oder polymorphe Schwangerschaftsdermatose (AEP/PEP), Pemphigoid gestationis
Bei unauffälligem Hautbefund: Basislabordiagnostik (s. oben) plus Gallensäuren (nüchtern)
**Mögliche weitergehende Untersuchungen**
Bei analem Pruritus: Parasiten, Wurmeier, digital-rektale Untersuchung, PSA
Bei aquagenem und genitalem Pruritus, Pruritus unklarer Genese: Laktose‑/Sorbit-Intoleranztest
Bei Blutbildveränderungen/Verdacht auf lymphoproliferative Erkrankungen: Vitamin B_12_, Folsäure, Eiweißelektrophorese, Immunfixation, JAK2-Status, ggf. KM-Punktion mit (Immun‑)Zytologie und Histologie
Bei Eisenmangel/Stuhlunregelmäßigkeiten: Stuhluntersuchung auf okkultes Blut
Bei pathologischen Leberwerten: Hepatitisserologie (Anti-HAV-IgM/IgG, HBsAg, Anti-HBc, Anti-HCV), ggf. direkte Virusnachweise HAV-RNA, HBV-DNA, HCV-RNA, HEV-RNA, Gallensäuren, antimitochondriale Antikörper (AMA), gp210, sp100, antinukleäre Antikörper (ANA), glatte Muskulatur-Antikörper (SMA), Soluble-liver-antigen-Antikörper (SLA), Liver-Kidney-mikrosomale Antikörper (LKM), Gewebstransglutaminase-AK, Alpha-Fetoprotein (bei Leberzirrhose/hepatischer Raumforderung)
Bei pathologischer Nüchternglukose: HBA_1c,_ Glukosetoleranztest
Bei primären oder sekundären Hautveränderungen: Direkte und indirekte Immunfluoreszenz, Autoantikörper gegen dermale Proteine (BP180, 230, Desmoglein)
Bei Verdacht auf Allergie: Gesamt-IgE, ggf. spezifische IgE, Prick-Testung, Epikutantestung
Bei Verdacht auf endokrine Erkrankungen: Parathormon, Phosphat, Ca^2+^, fT3, fT4, 25-OH-Cholecalciferol, TSH-Rezeptor-AK (TRAK), Thyreoperoxidase-AK (TPO-AK)
Bei Verdacht auf HIV: HIV-Serologie, ggf. Luesserologie
Bei Verdacht auf Mastozytose: Tryptase
Bei Verdacht auf neuroendokrine Tumoren: Chromogranin A
24-h-Sammelurin: Porphyrine (Porphyrien), 5‑Hydroxyindolessigsäure (neuroendokrine Tumoren), Methylimidazolessigsäure (Mastozytose)
*Bildgebende Verfahren* (auch wenn in der Anamnese, bei der körperlichen Untersuchung und der Labordiagnostik kein spezifischer Krankheitsverdacht besteht, können ein Röntgenbild bzw. eine Computertomographie des Thorax und eine Sonographie des Abdomens veranlasst werden, um Hinweise auf eine evtl. bestehende maligne Erkrankung gewinnen zu können)
*Interdisziplinäre Kooperationen*
Kooperation mit weiteren (Fach‑)Ärztinnen/en: Allgemeinmedizin, Allergologie, Dermatologie, Innere Medizin (Gastroenterologie, Hepatologie, Endokrinologie, Hämatologie und internistische Onkologie), Urologie, Gynäkologie etc.
Neurologischer und/oder psychiatrischer Fachbefund

*AEP* atopische oder polymorphe Schwangerschaftsdermatose, *ALAT* Alanin-Amino-Transaminase, *AMA* Antimitochondriale Antikörper, *ANA* Antinukleäre Antikörper, *ASAT* Aspartat-Amino-Transaminase, *BSG* Blutsenkungsgeschwindigkeit, *CRP* C-reaktives Protein, *eGFR* errechnete glomeruläre Filtrationsrate, *HAV* Hepatitis-A-Virus, *HBV* Hepatitis-B-Virus, *HCV* Hepatitis-C-Virus, *HEV* Hepatitis-E-Virus, *HIV* Humanes Immundefizienz Virus, *IgE* Immunglobulin E, *JAK2* Januskinase 2, *LDH* Laktatdehydrogenase, *LKM* Liver-Kidney-Mikrosomale Antikörper, *PEP* polymorphe Schwangerschaftsdermatose, *SMA* glatte Muskulatur-Antikörper, *SLA* soluble liver antigen-Antikörper, *TPO-AK* Thyreoperoxidase-Antikörper, *TRAK* TSH-Rezeptor-Antikörper, *TSH* Thyroidea-stimulierendes Hormon

**Figure 1:**
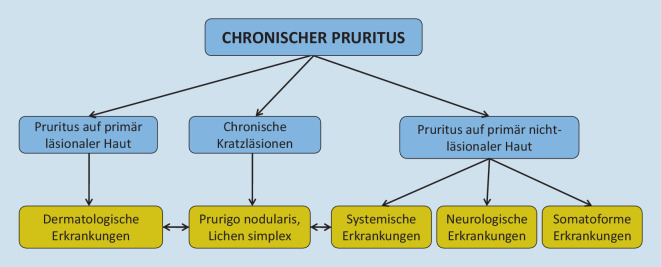

Pruritus auf primär nichtläsionaler Haut wird häufig durch systemische, neurologische und somatoforme Erkrankungen oder eine Medikamenteneinnahme ausgelöst (Tab. 2). Anamnese und klinische Untersuchung erbringen hierbei häufig wichtige Hinweise (Abb. 1 und 2). Laborchemisch wird die in Tab. 1 aufgezeigte Stufendiagnostik empfohlen, die sich an der Häufigkeit der mit Pruritus assoziierten Erkrankungen orientiert. Die Basisdiagnostik ermöglicht insbesondere auf hausärztlicher Ebene eine kostengünstige und gezielte Abklärung für die häufigsten internistischen Ursachen des chronischen Pruritus. Auffällige Befunde sollten durch mögliche weiterführende Untersuchungen abgeklärt werden (Tab. 1). Es sei angemerkt, dass neurologische oder psychiatrische Erkrankungen sowie in seltenen Fällen auch manche Tumorerkrankungen wie etwa Prostatakarzinome, lymphoproliferative Neoplasien, gastrointestinale Malignome oder kleine, neuroendokrine Tumoren unentdeckt bleiben können. In 2 großen Kohortenstudien mit 8744 und 12.813 Patienten konnten bei der Abklärung des chronischen Pruritus auf primär nichtläsionaler Haut erhöhte Malignomraten allerdings nur bei hämatologischen Erkrankungen, insbesondere Lymphomen und lymphoproliferative Neoplasien, sowie Gallengangskarzinomen nachgewiesen werden [9, 14].

**Table 2:** 

Organsystem	Erkrankungsbeispiele
Renale Erkrankungen	Chronische Niereninsuffizienz jeglicher Genese
Hepatobiliäre Erkrankungen	Primär biliäre Cholangitis, primär/sekundär sklerosierende Cholangitis, IgG4-assoziierte Cholangiopathie, medikamentöse/toxische intrahepatische Cholestase, Alagille-Syndrom, familiäre Cholestasesyndrome (BRIC, PFIC), intrahepatische Schwangerschaftscholestase, Leberzirrhose jeglicher Genese, obstruktive Cholestase jeglicher Genese wie Gallengangssteine, -adenome, -karzinome, Pankreaskopfkarzinome
Hämatopoetische Erkrankungen	Polycythaemia vera, essenzielle Thrombozythämie, myelodysplastisches Syndrom, Morbus Hodgkin, Non-Hodgkin-Lymphome, Hypereosinophiliesyndrome, Mastozytose
Endokrine Erkrankungen	Hypo‑/Hyperthyreose, Hyperparathyreoidismus, Karzinoidsyndrom, Diabetes mellitus
Malassimilationssyndrome	Laktoseintoleranz, Zöliakie, Anorexia nervosa, Eisenmangel, Vitamin-B/D-Mangel
Infektionskrankheiten	Akute/chronische HBV-/HCV-/HIV-/HSV-Infektionen, H.p.-Infektionen, VZV-Reaktivierung (postherpetische Neuralgie), Parasitosen
Solide Tumoren	Karzinome der Schilddrüse, des HNO-Bereichs, der Mamma, Lunge, des Magens, Pankreas, Kolons/Rektums, der Prostata, des Uterus und Sarkome

*Ig* Immunglobulin, *BRIC* benigne wiederkehrende intrahepatische Cholestase, *PFIC *progressive familiäre intrahepatische Cholestase, *HBV* Hepatitis-B-Virus, *HCV* Hepatitis-C-Virus, *HIV* humaner Immundefizienzvirus, *HSV* Herpes-simlex-Virus, *H.p. Helicobacter pylori, VZV* Varicella-Zoster-Virus, *HNO* Hals-Nasen-Ohren

**Figure 2:**
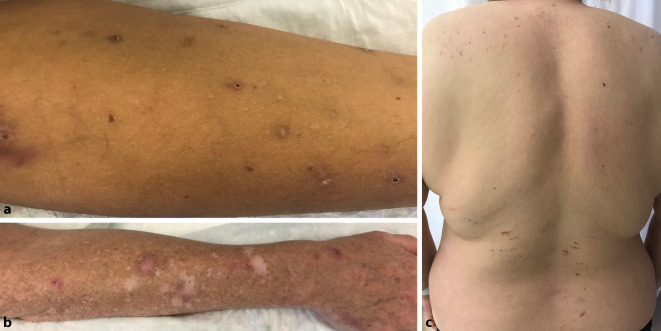

Trotz intensiver Abklärungen kann die Ursache des chronischen Pruritus bei einem Teil der Patienten ungeklärt bleiben. Je nach Literaturangabe und untersuchtem Kollektiv betrifft dies 13–50 % der Fälle [18]. Da Pruritus bei einigen Patienten als prämonitorischer Pruritus teils Monate bis Jahre vor dem Auftreten der auslösenden Erkrankung auftritt, kann eine erneute Kontrolle der Basisdiagnostik daher nach 6 bis 12 Monaten bzw. abhängig vom klinischen Verlauf erwogen werden.

## Pruritus bei chronischer Niereninsuffizienz

Während Patienten mit akutem Nierenversagen und Patienten mit Nierenerkrankungen ohne Einschränkung der Nierenfunktion nicht an Pruritus leiden, sind solche mit chronischer Niereninsuffizienz häufig betroffen. Patienten mit höhergradig eingeschränkter chronischer Nierenerkrankung, die noch keiner Dialysebehandlung bedarf, leiden ebenfalls zu einem erheblichen Prozentsatz unter chronischem Pruritus. Diese Form wird als „chronic kidney disease“-assoziierter Pruritus (CKD-aP) bezeichnet. Laut der sehr großen, multinationalen DOPPS-Kohorte mit über 35.000 Dialysepatienten leiden über 40 % der Dialysepatienten an mäßigem bis sehr starkem Pruritus [16]. Patienten an der Peritonealdialyse sind etwas seltener betroffen als Patienten an der Hämodialyse. Bezüglich der Lokalisation berichten Patienten häufig über Pruritus an Kopf und Rücken, 25–50 % klagen über einen generalisierten Pruritus.

Rund 40 % der Dialysepatienten leiden an mäßigem bis sehr starkem Pruritus

Die Therapie des CKD-aP ist aufgrund des limitierten Wissensstandes über die pathophysiologischen Veränderungen und zugrunde liegenden Signalwege weitgehend empirisch. Zum Teil erbrachten verfügbare Therapiestudien widersprüchliche Resultate. Zunächst sollte bei CKD-aP die Dialyseeffizienz überprüft und ggf. optimiert werden. Weiterhin gilt es, die Parameter des sekundären Hyperparathyreoidismus (d. h. Kalzium, Phosphat, Parathormon, Vitamin D) zu kontrollieren und ggf. therapeutisch in die jeweiligen Zielbereiche zu korrigieren. Als Basistherapie sowie bei milder Pruritusintensität werden zunächst topische Therapien empfohlen. In klinischen und Beobachtungsstudien beeinflussten Capsaicin‑, Gammalinolensäure- und Tacrolimus-haltige Cremes insbesondere milden CKD-aP günstig. Bei moderatem bis schwerwiegendem Pruritus können die Antiepileptika, wie z. B. Gabapentin (initial 100 mg per os an Hämodialysetagen, je nach Ansprechen und Verträglichkeit bis zu maximal 300 mg an Hämodialysetagen), gegeben werden. Alternativ kann auch Pregabalin eingesetzt werden (initial 50 mg per os an Hämodialysetagen, je nach Ansprechen und Verträglichkeit bis zu maximal 75 mg täglich). Bei diesen beiden Therapien handelt es sich allerdings um Off-label-Therapien (Tab. 3; [28]). Mehrere randomisierte, placebokontrollierte Kurzzeitstudien unterstreichen einen positiven, vergleichbaren Effekt für beide Substanzen [7]. Aufgrund der rein renalen Elimination beider Medikamente sollten diese in niedriger Dosierung eingeschlichen werden. Mögliche Nebenwirkungen sind Benommenheit, Schwindel und v. a. bei älteren Patienten eine erhöhte Sturzgefahr mit Frakturrisiko [13]. Die publizierten Daten zum µ‑Opioid-Rezeptor-Antagonisten Naltrexon sind widersprüchlich, einschleichende Dosierungen beginnend mit 25 mg/Tag können im Einzelfall aber erwogen werden (Tab. 3). Antihistaminika weisen keinen Nutzen jenseits des Placeboeffekts auf. Insbesondere ältere, sedierende Antihistaminika sollten aufgrund der zunehmenden Fatigue und Tagesmüdigkeit vermieden werden [25].

**Table 3:** 

Medikament^a^	Dosierung
Difelikefalin	0,5 µg/kgKG an HD-Tagen (i.v.)
Gabapentin^a, b^	Initial 100 mg an HD-Tagen (p.o.)
Pregabalin^a, b^	Initial 50 mg an HD-Tagen (p.o.)
UVB-Lichttherapie	1- bis 2‑mal/Woche
Naltrexon^a, c^ Naloxon^a, c^	25–50 mg/Tag (p.o.) 0,002–0,2 μg/kgKG/min (ggf. 0,4 mg Bolus)
Experimentelle Behandlungen/klinische Studien	–

*i.v.* intravenös, *KG* Körpergewicht, *HD* Hämodialyse, *p.o.* per os

^a^ Nur Difelikefalin ist zugelassen, alle anderen Medikamente fallen unter den Off-label-Use

^b^ Dosis einschleichen; cave: insbesondere bei älteren Patienten/innen Schwindel und Sturzgefahr

^c^ Cave: mögliche Opioid-ähnliche Entzugssymptomatik; daher einschleichende Dosierung, ggf. mittels Naloxon-Perfusor mit sukzessiver Dosissteigerung und Oralisierung

Neuere Therapieansätze basieren auf einer postulierten verstärkten µ‑Opioid-Rezeptor(MOR)- und verminderten κ‑Opioid-Rezeptor(KOR)-Aktivierung bei Patienten mit CKD-aP. Mit dem Ziel, dieses Ungleichgewicht wieder in Einklang zu bringen, sind mehrere KOR-Agonisten entwickelt worden. Während der zentralwirksame KOR-Agonist Nalfurafin aufgrund seines geringen therapeutischen Effekts nur in Japan zugelassen ist, wurde kürzlich der peripher wirkende KOR-Agonist Difelikefalin von der Food and Drug Administration (FDA) als erstes Medikament zur spezifischen Behandlung des CKD-aP zugelassen [10]. Difelikefalin, das 3‑mal/Woche intravenös nach der jeweiligen Dialysebehandlung appliziert wurde, zeigte in einer Phase-III-Studie mit 378 Hämodialysepatienten eine signifikante Reduktion der Pruritusintensität im Vergleich zu der Placebogruppe. In April 2022 wurde Difelikefalin auch in Europa von der European Medicines Agency (EMA) zur Therapie zugelassen.

Nichtmedikamentöse Behandlungsoptionen sind die ultraviolette Lichttherapie (UVB), Akupunktur oder Elektroakupunktur (Tab. 3). Nach einer erfolgreichen Nierentransplantation sistiert meist der Pruritus.

## Pruritus bei Leber- und Gallenwegserkrankungen

Patienten mit hepatobiliären Erkrankungen berichten häufig über chronischen Pruritus. Klassischerweise sind Patienten mit laborchemischer Cholestase betroffen, neuere Daten belegen aber auch ein Auftreten ohne Cholestase wie bei Fettlebererkrankung oder Leberzirrhose. Die Prävalenz des hepatischen Pruritus variiert je nach zugrunde liegender Erkrankung. Als definierendes Symptom tritt er bei der intrahepatischen Schwangerschaftscholestase auf. Sehr häufig findet sich Pruritus auch bei der benignen wiederkehrenden intrahepatischen Cholestase (BRIC) sowie deren progressiven familiären Formen (PFIC). Patienten mit primär biliärer Cholangitis (PBC) sowie primär bzw. sekundär sklerosierender Cholangitis (PSC/SSC) leiden im Krankheitsverlauf in bis zu 70 % der Fälle an Pruritus, Patienten mit benignen und maligen extrahepatischen Gallengangsveränderungen in 15–45 % und solche mit chronischer Hepatitis-C-Virus(HCV)-Infektion in 5–15 %.

Hepatischer Pruritus kann v. a. bei Patienten mit PBC und PSC an den Extremitäten lokalisiert sein, charakteristisch sind dabei betroffene Handinnenflächen und Fußsohlen. In vielen Fällen wird Pruritus aber im Krankheitsverlauf als generalisiert berichtet [4]. Patientinnen beschreiben teils ein intensiviertes Juckempfinden prämenstruell, während Hormonersatztherapien und im letzten Trimenon einer Schwangerschaft. Pruritus bei hepatobiliären Erkrankungen ist unabhängig vom Ausmaß der Cholestase, tritt häufig bereits in frühen Krankheitsstadien oder prämonitorisch auf, also vor Diagnosestellung.

Die Behandlungsmöglichkeiten beschränken sich auf wenige evidenzbasierte und einige experimentelle medikamentöse und interventionelle Therapien. Therapieansätze sollten primär auf die adäquate Behandlung der zugrunde liegenden Erkrankung fokussieren, da sich hierdurch der Pruritus häufig bessert oder zurückbildet. Pruritus aufgrund einer extrahepatischen Obstruktion des Gallengangssystems bessert sich häufig durch eine endoskopische Dilatation ggf. mit Stentimplantation, perkutaner oder nasobiliärer Drainage. Pruritus bei intrahepatischer Cholestase benötigt dagegen häufig eine systemische Therapie und kann therapeutisch herausfordernd sein.

Milder Pruritus, Xerosis und Hautläsionen können effektiv mittels rehydratisierender, rückfettender und ggf. kühlender, z. B. mentholhaltiger Topika behandelt werden. Wenngleich Ursodeoxycholsäure (UDCA) die Basistherapie vieler cholestatischer Erkrankungen wie PBC, PSC, Schwangerschaftscholestase und pädiatrischer Cholestasesyndrome darstellt [3], bessert dieses Medikament lediglich den Pruritus bei intrahepatischer Schwangerschaftscholestase [11]. Antihistaminika werden im klinischen Alltag weiterhin häufig eingesetzt. Allerdings weisen sie keinen Nutzen jenseits des Placeboeffekts auf und sind daher nicht empfohlen.

Therapieansätze sollten primär auf adäquate Behandlung der zugrunde liegenden Erkrankung fokussieren

Das Antibiotikum Rifampicin erwies sich in mehreren randomisierten, placebokontrollierten Studien als wirksam [22]. Es sollte in einer einschleichenden Dosierung von 150 mg/Tag eingesetzt werden, da in vielen Fällen bereits 150–300 mg/Tag effektiv den Pruritus lindern. Wichtig ist, das Interaktionspotenzial mit anderen Medikamenten wie orale Antikoagulanzien, orale Kontrazeptiva, Immunsuppressiva oder Antiepileptika zu beachten. Eine Hepatotoxizität wird in etwa 5 % der Fälle in der Langzeitanwendung beobachtet. Daher sollten die Transaminasen nach 2, 6 und 12 Wochen sowie nach einer Dosisänderung kontrolliert werden [24] Aufgrund einer Eigenfarbe des Medikaments verfärben sich Körperflüssigkeiten orange-rötlich unter einer Rifampicin-Therapie, worauf Patienten hingewiesen werden sollten.

Bezafibrat wirkt nicht nur anticholestatisch, sondern weist auch antipruriginöse Effekte auf. In einer 3‑wöchigen randomisierten, placebokontrollierten Studie verbesserte Bezafibrat in einer Dosierung von 400 mg/Tag die Juckintensität um mindestens 50 % bei knapp der Hälfte der eingeschlossenen PBC-, PSC- und SSC-Patienten [6]. Die häufigste Nebenwirkung ist eine Myopathie, die in etwa 20 % der Fälle beobachtet wird. Ferner trat in der BEZURSO-Studie bei 6 % der Patienten eine Hepatotoxizität auf, die teils einer Glukokortikoid-Therapie bedurfte [5]. Daneben sollte Bezafibrat aufgrund einer möglichen Nephrotoxizität nicht bei Patienten mit einer errechneten glomerulären Filtrationsrate (eGFR) unterhalb von 60 ml/min verschrieben werden. Für Patienten mit dekompensierter Leberzirrhose liegen bisher keine Daten vor, weshalb diese nicht behandelt werden sollten.

Eine weitere Option stellen die MOR-Antagonisten Naltrexon und Naloxon dar. Mehrere randomisierte, placebokontrollierte Studien haben einen, wenngleich geringen Vorteil über Placebo erbracht [22]. Aus klinischer Erfahrung ist der Nutzen dieser Substanzen gering, allerdings kann im stationären Setting eine intravenöse Naloxon-Infusion mit konsekutiver Dosissteigerung insbesondere bei fortgeschrittenen Krankheitsstadien erfolgreich sein. Aufgrund einer möglichen Opiat-ähnlichen Entzugssymptomatik sollten diese Substanzen in einschleichender Dosierung angewandt werden (Tab. 4). Um einem Gewöhnungseffekt vorzugbeugen, kann es hilfreich sein, die Medikation an 1 oder 2 Tagen pro Woche zu pausieren. Die selektiven Serotoninwiederaufnahmeinhibitoren Sertralin und Paroxetin können ebenfalls als antipruriginöse Therapie bei hepatischem Pruritus erwogen werden.

**Table 4:** 

Medikament^a^	Dosierung
Colestyramin^a, b^	4–16 g/Tag (p.o.)
Rifampicin^a, c^	150–600 mg/Tag (p.o.)
Bezafibrat^a, d^	200–400 mg/Tag (p.o.)
Naltrexon^a, e^ Naloxon^a, e^	25–50 mg/Tag (p.o.) 0,002–0,2 μg/kgKG/min (ggf. 0,4 mg Bolus)
Gabapentin^a, f^	100–3600 mg/Tag (p.o.)
Pregabalin^a, f^	25–600 mg/Tag (p.o.)
Sertralin	75–100 mg/Tag (p.o.)
UVB-Lichttherapie	1- bis 2‑mal/Woche
Experimentelle Behandlungen/klinische Studien	–

*p.o.* per os, *KG* Körpergewicht

^a^ Nur Colestyramin ist für die Behandlung des cholestatischen Pruritus zugelassen; alle anderen Medikamente fallen unter den Off-label-Use

^b^ Cave: Einnahme anderer Medikamente mit zeitlichem Abstand von 4 h

^c^ Häufig sind 150–300 mg/Tag ausreichend. Cave: Interaktionspotenzial; bei Langzeitbehandlung Hepatotoxizität in etwa 5 %: laborchemische Kontrolle nach 2, 6 und 12 Wochen sowie Dosisänderung

^d^ Cave: Dosisreduktion bei eingeschränkter Nierenfunktion; kontraindiziert bei Dialyse; Myopathie sowie erhöhte Rhabdomyolysegefahr bei gleichzeitiger Statineinnahme; bei Langzeitbehandlung Hepatotoxizität in etwa 5 %: laborchemische Kontrolle nach 2, 6 und 12 Wochen sowie Dosisänderung

^e^ Cave: einschleichende Dosierung, um Nebenwirkungen zu reduzieren, ggf. mittels Naloxon-Perfusor mit sukzessiver Dosissteigerung und Oralisierung

^f^ Bei älteren Patienten/innen und eingeschränkter Nierenfunktion Dosis einschleichen/anpassen (s. auch Dosierung bei CKD[„chronic kidney disease“]-assoziiertem Pruritus)

Eine wichtige zukünftige therapeutische Option sind Inhibitoren des ilealen Gallensalztransporters (IBAT), die selektiv die Rückresorption von Gallensäuren aus dem Darmlumen in den Enterozyten unterbrechen. Aufgrund der positiven Daten der Phase-III-Studien PEDFIC‑1 und -2 wurde der IBAT-Inhibitor Odevixibat (Bylvay^TM^, Albireo Pharma, Boston, MA, US) bereits im Juli 2021 durch die FDA zur Behandlung des hepatischen Pruritus bei PFIC-Kindern ab dem 3. Lebensmonat zugelassen (bisher nicht als Manuskript veröffentlicht). Die eindrucksvollen Ergebnisse der ICONIC-Studie führten ebenfalls zur FDA-Zulassung von Maralixibat (Livmarli^TM^, Mirum Pharmaceuticals, Foster City, CA, USA) im Herbst 2021 zur Behandlung des hepatischen Pruritus bei Kindern mit Alagille-Syndrom ab dem 1. Lebensjahr [12]. Weitere IBAT-Inhibitoren wie Linerixibat und Volixibat befinden in Phase-II- und -III-Studien. Das Nebenwirkungsprofil basiert insbesondere auf der enteralen Ausscheidung von Gallensäuren mit entsprechender gastrointestinaler Symptomatik sowie teils relevanter und therapielimitierender Diarrhö. Die Substanz Elobixibat nutzt allerdings diesen Effekt als therapeutische Anwendung bei chronischer Obstipation und wurde hierfür in Japan zugelassen.

Bei unzureichendem Therapieansprechen dieser medikamentösen Therapieoptionen können interventionelle Verfahren wie Plasmapherese, Albumindialyse, transkutane oder nasobiliäre Drainage eingesetzt werden. Nach erfolgreicher Lebertransplantation sistiert meist der hepatische Pruritus.

## Pruritus bei hämatoonkologischen Erkrankungen

Auch hämatologische Erkrankungen können die Ursache eines generalisierten Pruritus unklarer Ursache sein. Eine prospektive Studie mit 95 Patienten mit Pruritus unklarer Ätiologie erbrachte bei 7 % aller Patienten eine bis dahin nicht diagnostizierte myeloproliferative Neoplasie [1].

Insbesondere Patienten mit Polycythaemia vera (PV), einer seltenen myeloproliferativen Erkrankung, berichten in bis zu 30–65 % über Juckempfinden. Häufig handelt es sich hierbei um einen aquagenen Pruritus mit stechendem Jucken nach Wasserkontakt, etwa nach dem morgendlichen Duschen. Am häufigsten sind Patienten mit einer homozygoten JAK2 617V-Mutation betroffen. Interessanterweise verbessern entsprechende Inhibitoren des überaktivierten JAK-STAT-Signalwegs wie Ruxolitinib nicht nur die zugrunde liegende Erkrankung, sondern lindern rasch auch den Pruritus [15, 23].

Im Gegensatz zur PV, bei der es sich um eine Erkrankung der myeloiden Blutzellreihe handelt, ist der Morbus Hodgkin eine lymphoproliferative Erkrankung. Patienten mit Morbus Hodgkin klagen in 15–50 % über Pruritus auf nichtläsionaler Haut [26]. Dies kann sogar Monate bis Jahre vor Ausbruch der Erkrankung oder einem Rezidiv auftreten. Patienten mit Non-Hodgkin-Lymphomen berichten mit bis zu 30 % etwas seltener über Jucken. Auch bei Leukämien kann chronischer Pruritus auftreten, dies ist häufiger mit lymphatischen als myeloiden und öfter mit chronischen als akuten Verlaufsformen assoziiert. Therapieempfehlungen bei Pruritus myeloproliferativer Neoplasien sind der Tab. 5 zu entnehmen.

**Table 5:** 

Medikament^a^	Dosierung
Gabapentin^a, b^	100–3600 mg/Tag (p.o.)
Pregabalin^a, b^	25–600 mg/Tag (p.o.)
Paroxetin^a^	20 mg/Tag (p.o.)
Acetylsalicylsäure^a^	300 mg/Tag (p.o.)
Mirtazapin^a^	7,5–30 mg/Tag (p.o.)
Naltrexon^a, c^	25–150 mg/Tag (p.o.)
UVB-Lichttherapie	1- bis 2‑mal/Woche
Experimentelle Behandlungen/klinische Studien	–

^a^ Alle Medikamente fallen unter den Off-label-Use

^b^ Bei älteren Patienten/innen und eingeschränkter Nierenfunktion Dosis einschleichen/anpassen (s. auch Dosierung CKD[„chronic kidney disease“]-assoziierter Pruritus)

^c^ Cave: einschleichende Dosierung, um Nebenwirkungen zu reduzieren, ggf. mittels Naloxon-Perfusor mit sukzessiver Dosissteigerung und Oralisierung

## Pruritus bei soliden Tumoren

In seltenen Fällen können solide Tumoren mit einem paraneoplastischen Pruritus assoziiert sein. Bisher sind die auslösenden Pruritogene unbekannt. Am häufigsten tritt diese Form des chronischen Pruritus bei gastrointestinalen Tumoren auf. Allerdings entwickelt nur ein sehr geringer Anteil der Patienten mit Pruritus unklarer Ätiologie („pruritus of unknown origin“ [PUO]) im Verlauf ein Malignom. In 2 großen Kohortenstudien wurden erhöhte Malignomraten lediglich für hämatologische Erkrankungen sowie das Gallengangskarzinom berichtet [14]. Eine weitergehende Malignomabklärung mittels abdomineller Sonographie oder weitergehenden bildgebenden Verfahren kann daher bei Patienten mit chronischem Pruritus unklarer Ätiologie erwogen werden.

## Pruritus bei metabolischen und endokrinen Störungen

Wenngleich der Diabetes mellitus häufiger für seine assoziierte diabetogene Polyneuropathie mit Parästhesien und teils Schmerzen bekannt ist, belegen neuere Daten, dass Pruritus auch von bis zu 27 % der Typ-II-Diabetiker berichtet wird. Häufig beschreiben Patienten dabei einen lokalisierten Pruritus v. a. am Rumpf [27]. Als Risikofaktoren gelten die Dauer des Diabetes mellitus und das Vorhandensein einer diabetogenen Polyneuropathie.

Ferner kann chronischer Pruritus bei einer Hyperthyreose vorkommen. In einer Untersuchung bestand Pruritus bei 11 % der Patienten mit Morbus Basedow, insbesondere waren solche mit thyreotoxischer Krise betroffen. Deutlich häufiger findet sich Pruritus bei Hypothyreose. Dieser Pruritus wird wahrscheinlich durch die Xerosis cutis ausgelöst und spricht häufig gut auf rehydrierende und rückfettende topische Therapien an. Ebenfalls kann Pruritus beim sekundären, gelegentlich auch primären Hyperparathyreoidismus auftreten.

Generalisierter Pruritus tritt auch bei Essstörungen wie der Anorexia nervosa auf und betrifft etwa jeden fünften Patienten [21]. Ursächlich ist möglicherweise die Xerosis cutis, die sich in etwa 60 % der Patienten findet. Es konnte zudem eine reverse Korrelation zwischen Body Mass Index (BMI) und Juckempfinden sowie eine deutliche Besserung nach Gewichtszunahme gezeigt werden.

Auch Malassimilationssyndrome sind mit chronischem Pruritus assoziiert. Interessanterweise konnte bei Patienten mit aquagenem Pruritus in 25 % der Fälle eine Laktoseintoleranz als möglicher Auslöser eruiert werden. Eine laktosefreie Diät linderte den Pruritus bei 64 % der Patienten. Ebenfalls berichten Patienten mit Gluten-sensitiver Enteropathie (Zöliakie) über generalisierten Pruritus. Als pathogenetische Faktoren werden in bestimmten Fällen auch die assoziierte PBC, die Dermatitis herpetiformis oder der im Rahmen der Zöliakie auftretende Eisenmangel diskutiert. Eisenmangel kann mit einem generalisierten oder lokalisierten Pruritus, insbesondere anogenital einhergehen. Dieser bessert sich oder sistiert unter Eisensubstitution.

## Pruritus bei Infektionserkrankungen

Bestimmte chronische Infektionen sind mit chronischem Pruritus assoziiert. So berichten viele Patienten mit einer humanen Immundefizienzvirus(HIV)-Infektion im Laufe ihrer Erkrankung über Pruritus, teils durch HIV-assoziierte Hauterkrankungen wie papulosquamöse Veränderungen, Hautinfektionen oder Xerosis. Aber auch in Abwesenheit primärer Hautveränderungen kann chronischer Pruritus das erste Symptom bei HIV-Infektion darstellen und daher bei Pruritus unbekannten Ursprungs ein HIV-Screening erwogen werden.

Herpes zoster stellt eine Reaktivierung des Varizella-Zoster-Virus dar und kann mit einer sehr schmerzhaften postherpetischen Neuropathie assoziiert sein. In einigen Fällen leiden Patienten dabei auch an schwerwiegendem Pruritus, der meist auf das betroffene Hautareal lokalisiert ist. Eine Zoster-Impfung reduziert nicht nur die Wahrscheinlichkeit einer Neuralgie, sondern sollte auch pruriginöse Erscheinungsbilder weitgehend verhindern.

Lokalisierter oder generalisierter Pruritus kann auch bei vielen weiteren parasitären wie viralen Infektionen mit Hepatitis-B- und -C-, Herpes-simplex- oder Dengue-Fieber-Viren auftreten. Pruritus ohne primär läsionale Hautveränderungen finden sich v. a. bei chronischen viralen Hepatitiden.

## Pruritus im Alter

Chronischer Pruritus ist ein häufiges Symptom beim alten Patienten und kann durch zahlreiche dermatologische und internistische Erkrankungen verursacht werden [2]. Allerdings kann Pruritus auch ohne zugrunde liegende Erkrankungen auftreten. Alternde Haut ist charakterisiert durch Atrophie, reduzierte Hautbarriere und Xerosis aufgrund eines geringeren Wassergehaltes. In einem Tiermodell war in alternder Haut insbesondere eine reduzierte Anzahl an den für das Tastempfinden relevanten Merkelzellen von pathophysiologischer Relevanz für verstärktes Kratzverhalten [8].

## Medikamenteninduzierter Pruritus

Medikamente sind eine wichtige Differenzialdiagnose bei chronischem Pruritus [17]. Prinzipiell kann jedes Medikament Pruritus verursachen. Häufige medikamentöse Auslöser sind in der aktuellen S2k-Leitlinie „Chronischer Pruritus“ übersichtlich aufgelistet. Wenngleich medikamentenassoziierter Pruritus häufig mit morbilliformen und urtikariellen Hauterscheinungen einhergeht, handelt es sich meist nicht um eine echte Allergie, sondern um eine pseudoallergische Reaktion. Diese wird nicht über Ig(Immunglobulin)E-Rezeptoren, sondern über den Mas-related G‑Protein-gekoppelten Rezeptor X2 (MRGPRX2) auf Mastzellen vermittelt. Dies gilt insbesondere auch für viele Antibiotika. Daneben induzieren einige Medikamente chronischen Pruritus auch über bisher unbekannte Signalwege ohne primäre Hauterscheinungen, teils mit einer deutlichen Latenzzeit.

## Fazit für die Praxis

- Bei chronischem Pruritus auf primär nichtläsionaler Haut sollten internistisch-nephrologische, neurologische und psychiatrische Erkrankungen sowie Medikamentennebenwirkungen in Betracht gezogen werden.
- Jucken Handinnenflächen oder Fußsohlen ohne primäre Läsionen, liegen ursächlich meist hepatobiliäre Erkrankungen vor.
- Aquagener Pruritus tritt häufig bei myeloproliferativen Neoplasien auf.
- Für die hausärztliche Praxis wird eine laborchemische Basisdiagnostik empfohlen.
- Rifampicin und Bezafibrat weisen den größten Nutzen bei hepatischem Pruritus auf.
- Als Off-label-Therapien lindern Gabapentin und Pregabalin in nierenadaptierter Dosierung den „chronic kidney disease“-assoziierten Pruritus (CKD-aP) in vielen Fällen.
- Difelikefalin zeigte bei Hämodialysepatienten sehr vielversprechende Studienergebnisse und wurde kürzlich sowohl von der Food and Drug Administration (FDA) als auch der European Medicines Agency (EMA) speziell für die medikamentöse Therapie des CKD-aP zugelassen.
- Bei myeloproliferativen Erkrankungen sind v. a. JAK-STAT-Inhibitoren günstig zur Therapie eines zugrunde liegenden Pruritus.

## Abkürzungen

BMI: Body Mass Index
BRIC: Benigne wiederkehrende intrahepatische Cholestase
CKD-aP: Chronic kidney disease-assoziierter Pruritus
eGFR: Errechnete glomeruläre Filtrationsrate
EMA: European Medicines Agency
FDA: Food and Drug Administration
HAV: Hepatitis-A-Virus
HBV: Hepatitis-B-Virus
HCV: Hepatitis-C-Virus
HD: Hämodialyse
HEV: Hepatitis-E-Virus
HIV: Humaner Immundefizienzvirus
H.p.: Helicobacter pylori
HSV: Herpes-simplex-Virus
IBAT: Ilealer Gallensalztransporter
Ig: Immunglobulin
JAK-STAT: Januskinase-„signal transducers and activators of transkription“
KG: Körpergewicht
KOR: κ‑Opioid-Rezeptor
MOR: µ‑Opioid-Rezeptor
MRGPRX2: Mas-related G‑Protein gekoppelter Rezeptor X2
PBC: Primär biliäre Cholangitis
PFIC: Progressive familiäre intrahepatische Cholestase
PSC: Primär sklerosierende Cholangitis
PUO: Pruritus unklarer Ätiologie
PV: Polycythaemia vera
SSC: Sekundär sklerosierende Cholangitis
UVB: Ultraviolettes B‑Licht
UDCA: Ursodeoxycholsäure
VZV: Varicella-Zoster-Virus
